# Copper and zinc content in wild game shot with lead or non-lead ammunition – implications for consumer health protection

**DOI:** 10.1371/journal.pone.0184946

**Published:** 2017-09-21

**Authors:** Daniela Schlichting, Christine Sommerfeld, Christine Müller-Graf, Thomas Selhorst, Matthias Greiner, Antje Gerofke, Ellen Ulbig, Carl Gremse, Markus Spolders, Helmut Schafft, Monika Lahrssen-Wiederholt

**Affiliations:** German Federal Institute for Risk Assessment, Berlin, Germany; University of Lleida, SPAIN

## Abstract

The aim of this study was to examine the contamination of game meat with copper and zinc and establish whether the use of alternative (non-lead) ammunition can lead to higher or unsafe levels of copper and zinc in the meat of roe deer, wild boar and red deer. The research project “Safety of game meat obtained through hunting” (LEMISI) was conducted in Germany with the purpose of examining the entry of lead as well as copper and zinc into the meat of hunted game when using either lead or non-lead ammunition.

The outcome of this study shows that the usage of both lead-based ammunition and alternative non-lead ammunition results in the entry of copper and zinc into the edible parts of the game. Using non-lead ammunition does not entail dangerously elevated levels of copper and zinc, so replacing lead ammunition with alternative ammunition does not introduce a further health problem with regard to these metals. The levels of copper and zinc in game meat found in this study are in the range found in previous studies of game. The content of copper and zinc in game meat is also comparable to those regularly detected in meat and its products from livestock (pig, cattle, sheep) for which the mean human consumption rate is much higher. From the viewpoint of consumer health protection, the use of non-lead ammunition does not pose an additional hazard through copper and zinc contamination. A health risk due to the presence of copper and zinc in game meat at typical levels of consumer exposure is unlikely for both types of ammunition.

## Introduction

Lead or non-lead, that is the question: whether lead ammunition for hunting can or should be replaced by non-lead ammunition–due to health concerns about lead levels in game meat—has been discussed intensely in recent years [1, 2]. Not only the question of a possible entry of lead into the edible parts of game meat through the different bullet types has been raised, but also whether the other metals used (i.e. copper and zinc) enter the meat in a similar way and if so, their possible relevance for consumer health protection [3–5].

Non-lead bullets are solid bullets made of copper or alloys of copper and zinc (tombac or brass), which—depending on their construction and impact velocity–either fragment or expand. Not much is known, however, about a possible increase of copper and zinc content in game meat through the use of non-lead bullets for hunting [4, 6].

In contrast to lead, copper and zinc are essential trace elements for humans. They are important parts of different enzymes, for example. Nonetheless, above a certain concentration, copper as well as zinc are also toxic according to Paracelsus’ observation that “the dose makes the poison”. Copper is stored in the liver and is excreted via the bile. Tolerable upper intake levels for copper are 1 to 5 mg per day, and for zinc 7 to 25 mg per day, depending on age [7].

In order to obtain a knowledge-based background for political decision making, the research project “Safety of game meat obtained through hunting (LEMISI)” was initiated [8]. The project was developed in six regions in Germany from 2011 to 2014 on behalf of the Federal Ministry of Food and Agriculture (BMEL). The effects of different bullet materials (lead versus non-lead) on the content of lead, copper and zinc in the edible parts of game meat were examined.

Within the scope of the LEMISI project, the influence of using alternative (non-lead) ammunition on the concentrations of copper and zinc in game meat was examined. The following questions were addressed in the course of the project:

1. Is there any difference in the copper and zinc content of the game meat between game hunted with lead ammunition compared to non-lead ammunition?
2. Is higher copper and zinc content measured in the area around the wound channel of animals killed with non-lead ammunition?
3. Are there significant differences in the copper and zinc content in the three subsamples taken from the edible tissue of hunted game (i.e., the area close to the wound channel, the saddle and the haunch)?

Previous experiences show that lead ammunition on average results in a higher lead content in game meat than non-lead ammunition [9, 10]. In the following, the data gained on the copper and zinc content in edible meat are presented and discussed in order to avoid replacing one problem with another.

## Material and methods

Within the scope of the study, samples of 1254 roe deer, 854 wild boar and 90 red deer from different regions within Germany were examined [8].

### Ethics statement

Licensed hunters killed the game analysed in this study during the established hunting season and in accordance with German regulations (German Hunting Act; Bundesjagdgesetz) and best practices. It did not involve any additional killing other than what is carried out in the German forests on a regular and managerial basis (population control). Permission was granted from the German Federal States (Länder) and their respective hunting authorities.

### Choice of regions

Within Germany six regions were chosen according to the lead content of the top soil in order to control lead concentrations attributable to soil lead contamination in the (statistical) analysis. Two regions were selected for each of the three lead levels in top soil (i.e. low lead content: < 30 mg lead/kg soil, medium lead content: 30 to 75 mg lead/kg soil and high lead content: > 75 mg lead/kg soil) chosen using a geographical map indicating lead content in top soil, thus resulting in a total of six regions [11]. The content of copper and zinc in soil were not taken into account due to the heterogeneity of soil conditions and the movement of animals.

### Experimental design and implementation

Quality assurance measures were integrated in all phases of the project. Hunters were instructed as to the aims of the research project. The animals were either shot with specific lead ammunition or with specific non-lead ammunition. For each animal killed, the hunters had to fill in a sample data sheet in which detailed information on the animals (species, age and gender) and how they had been shot (including bullet material, i.e. lead vs non-lead), bullet type used, information on the entry and exit of the bullet, shooting distance, bone hit (i.e. if the animal was killed by a shot that the hunter reported to have struck not only tissue and organs but also skeletal structures such as the ribs, scapula) were recorded. Parameters included in the statistical analysis were the animal species and bullet material—lead ammunition versus non-lead ammunition. The entry and exit of the bullet were considered in order to discuss the distribution of the metals in the meat depending on the place of entry. In addition, so called bone-hits (see above) were also examined. Here, the underlying hypothesis is that the resistance of the bone could lead to a further distribution of the metals in the muscle compared to bullet hits of “softer tissues”. The sample data sheet was also a vital part of the overall quality and assurance control (see below).

The hunted game was brought to game traders who had also been specifically trained for this project and who collected the samples according to uniform standards. Three samples were taken from each animal after completion of the regular process of skinning and cleaning the carcass according to hygiene standards for game meat [12]. The samples were taken from marketable meat of the saddle, haunch and the area close to the wound channel, which had been widely cut out. The sample amount was 100 g for each of the three subsamples. Subsamples were stored in coloured vials (i.e. one colour for each type of subsample). Samples were numbered and coded. All three subsamples per animal were stored in vials in polythene bags. The corresponding sample data sheet (with the identical coding) was stored in a separate polythene bag. These two bags were stored together in a third polythene bag so that it was possible to trace back each subsample to the location where the animal was shot, the laboratory where analyses were conducted and all the other relevant parameters given in the sample data sheet. In this way, this system served as quality assurance and control (i.e. plausibility check). Until the time of chemical analysis, samples were frozen and stored in polythene bags at −18 C.

### Analytics

The samples were transported to 12 accredited laboratories for chemical analysis: 11 of them from governmental agencies and one belonging to a leading international group of laboratories.

Before the beginning of chemical analysis, the samples were homogenized and 0.5 to 1g of each sample was put in a high-pressure Teflon container for microwave pressure digestion in line with EN 13805:2014 [13]. The content of copper in muscle samples was determined either by using the inductively coupled plasma–mass spectrometric method (ICP-MS), by applying inductively coupled plasma optical emission spectrometry (ICP-OES) or alternatively, by applying graphite furnace atomic absorption spectrometry (GFAAS) [14–16]. The zinc content in muscle samples was determined either with ICP-MS/ICP-OES or alternatively, by applying flame atomic absorption spectrometry (FAAS) [17].

### Determination of plausibility

The analytical results were sent to the Eberswalde University for Sustainable Development (Hochschule für nachhaltige Entwicklung Eberswalde, HNEE) for a plausibility check of the hunting and bullet data using the numeric coding of samples from the laboratories and the complete information from the data sheets. The most important item was the correct identification of the bullets used as reported by the hunters in the sample data sheets as “lead” or “non-lead”. The approved data were subsequently sent to the German Federal Institute for Risk Assessment (Bundesinstitut für Risikobewertung, BfR) where the statistical data analyses as well as the toxicological risk assessment were performed.

### Statistical evaluation

The copper and zinc content were quantifiable in all examined subsamples. Since the data were not distributed normally and the distributions were highly heterogeneous, group comparisons were done using non-parametric methods [18]. The Mann-Whitney U test was applied when comparing lead shot samples with non-lead shot samples. The comparison of the subsamples was made by applying either the Friedman test or the Wilcoxon signed-rank test. The significance level was determined as p<0.05. When comparing the subsamples, multiple testing was taken into account using a corresponding Bonferroni-adjusted significance level (p<0.017) [19].

The distribution of the analytical results is displayed graphically using beanplots (R-package “beanplot” [20]). Beanplots constitute an alternative to boxplots. They combine a density shape with a one-dimensional scatter plot–showing all analytical data as small lines–thereby allowing a visual comparison of the distribution [21].

Statistical analysis were realized using SPSS (IBM SPSS Statistics for Windows, Version 21.0). Corresponding graphs were created using R [22].

## Results

### Copper

**Fig 1:**
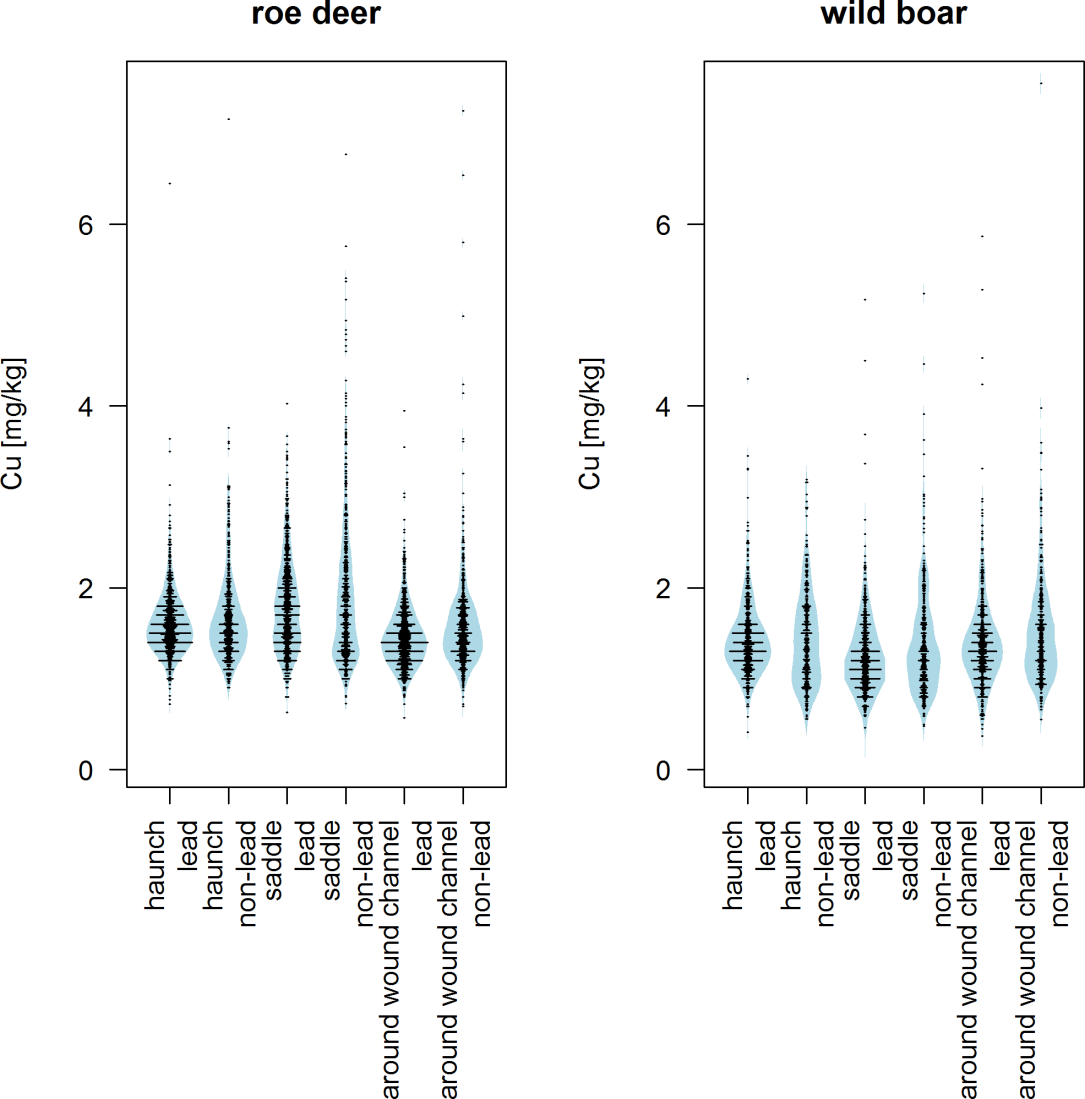
Copper content in different edible parts of roe deer and wild boar by bullet material (lead, non-lead).

The major part of the observed copper content in roe deer, wild boar and red deer which had been hunted using non-lead ammunition was in a low range. This fact is underlined by the height of the 95th percentiles (Table 1), as well as by the distribution of copper content in the beanplots (Fig 1 and S1 Fig).

**Table 1:** Copper content in hunted roe deer, wild boar and red deer (mg/kg).

Sample	Bullet	N	Mean^a^	Median	95th^b^	Maximum	P
Roe deer, haunch	Lead	745	1.614	1.564	2.196	6.451	0.359
	Non-lead	509	1.695	1.577	2.702	9.048	
Roe deer, saddle	Lead	745	1.810	1.759	2.769	4.034	0.576
	Non-lead	509	2.017	1.730	3.672	37.537	
Roe deer, around wound channel	Lead	745	1.464	1.400	2.063	3.946	<0.0001
	Non-lead	509	1.635	1.500	2.444	9.701	
Wild boar, haunch	Lead	514	1.437	1.375	2.136	4.300	0.432
	Non-lead	340	1.456	1.368	2.363	8.050	
Wild boar, saddle	Lead	514	1.506	1.200	1.986	110.000	0.005
	Non-lead	340	1.404	1.270	2.420	5.238	
Wild boar, around wound channel	Lead	514	1.426	1.322	2.286	9.616	0.005
	Non-lead	340	1.627	1.419	2.728	18.886	
Red deer, haunch	Lead	64	1.891	1.857	2.648	2.969	0.954
	Non-lead	26	1.896	1.874	2.478	2.902	
Red deer, saddle	Lead	64	1.794	1.746	2.462	4.787	0.789
	Non-lead	26	1.759	1.760	2.280	2.390	
Red deer, around wound channel	Lead	64	1.701	1.743	2.165	2.553	0.712
	Non-lead	26	1.755	1.650	2.363	2.721	

^a^ Arithmetical mean.

^b^ 95^th^ percentile.

The average copper content of the samples of non-lead shot roe deer was higher than that of lead shot roe deer. Thus the copper content close to the wound channel was significantly different depending on the type of ammunition (Mann-Whitney U test: p<0.0001; Table 1). But the samples from the area close to the wound channel of non-lead shot roe deer showed significantly lower copper content than samples from the haunch or saddle (Wilcoxon signed-rank test each: p<0.0001; Table 1). For the roe deer samples, the highest copper content was detected in a sample of the saddle (Table 1).

Overall, samples of 14 roe deer had copper content above 5 mg/kg. Thereof, 13 roe deer were shot with non-lead ammunition. Twelve of these animals were killed with a “bone hit” (for definition see material and methods section). One animal shot with non-lead ammunition and killed with bone hit had increased copper content in samples both from the area around the wound channel (9.70 mg/kg) and from the haunch (9.05 mg/kg).

For wild boar, the samples from the area close to the wound channel and the saddle showed significantly higher copper content when non-lead ammunition had been used (Mann-Whitney U test each: p = 0.005). Nevertheless, the highest copper content in wild boar samples was found in a sample from the saddle of an animal which had been shot with lead ammunition (Table 1 and Fig 1).

When using non-lead ammunition, the copper content in the area close to the wound channel in wild boar samples was higher than that of the haunch (Wilcoxon signed-rank test: p = 0.002) or saddle (Wilcoxon signed-rank test: p<0.0001). For lead shot animals, the samples from the area close to the wound channel showed significantly higher copper content than samples from the saddle (Wilcoxon signed-rank test: p<0.0001), but they were still below the copper content of the samples from the haunch (Wilcoxon signed-rank test: p = 0.008).

The copper content of a total of 12 wild boar samples was above a value of 5 mg/kg. Of these, four animals were shot using non-lead ammunition and seven animals using lead ammunition. From these animals, eight (nine samples) had been killed by a bone hit (non-lead: five samples; lead: four samples). In one animal which been shot using non-lead ammunition, the sample from the haunch as well as the sample from the area close to the wound channel had increased copper values (haunch 8.05 mg/kg and area close to the wound channel 7.55 mg/kg, bone hit).

The comparison of the copper content for red deer showed no significant differences between the use of non-lead or lead ammunition (Table 1).

A comparison between roe deer and wild boar showed that the copper content of roe deer was higher than that of wild boar (Mann-Whitney U test; Table 2) irrespective of the subsample and type of ammunition used.

**Table 2:** Differences in copper content of different tissues from roe deer and wild boar by bullet material (Mann-Whitney U test).

Sample	Bullet	Species	N	Mean^a^	Median	P
Haunch	Lead	Roe deer	745	1.614	1.564	<0.0001
		Wild boar	514	1.437	1.375	
	Non-lead	Roe deer	509	1.695	1.577	<0.0001
		Wild boar	340	1.456	1.368	
Saddle	Lead	Roe deer	745	1.810	1.759	<0.0001
		Wild boar	514	1.506	1.200	
	Non-lead	Roe deer	509	2.017	1.730	<0.0001
		Wild boar	340	1.404	1.270	
Around wound channel	Lead	Roe deer	745	1.464	1.400	<0.0001
		Wild boar	514	1.426	1.322	
	Non-lead	Roe deer	509	1.635	1.500	0.0010
		Wild boar	340	1.635	1.419	

^a^ Arithmetical mean.

### Zinc

**Fig 2:**
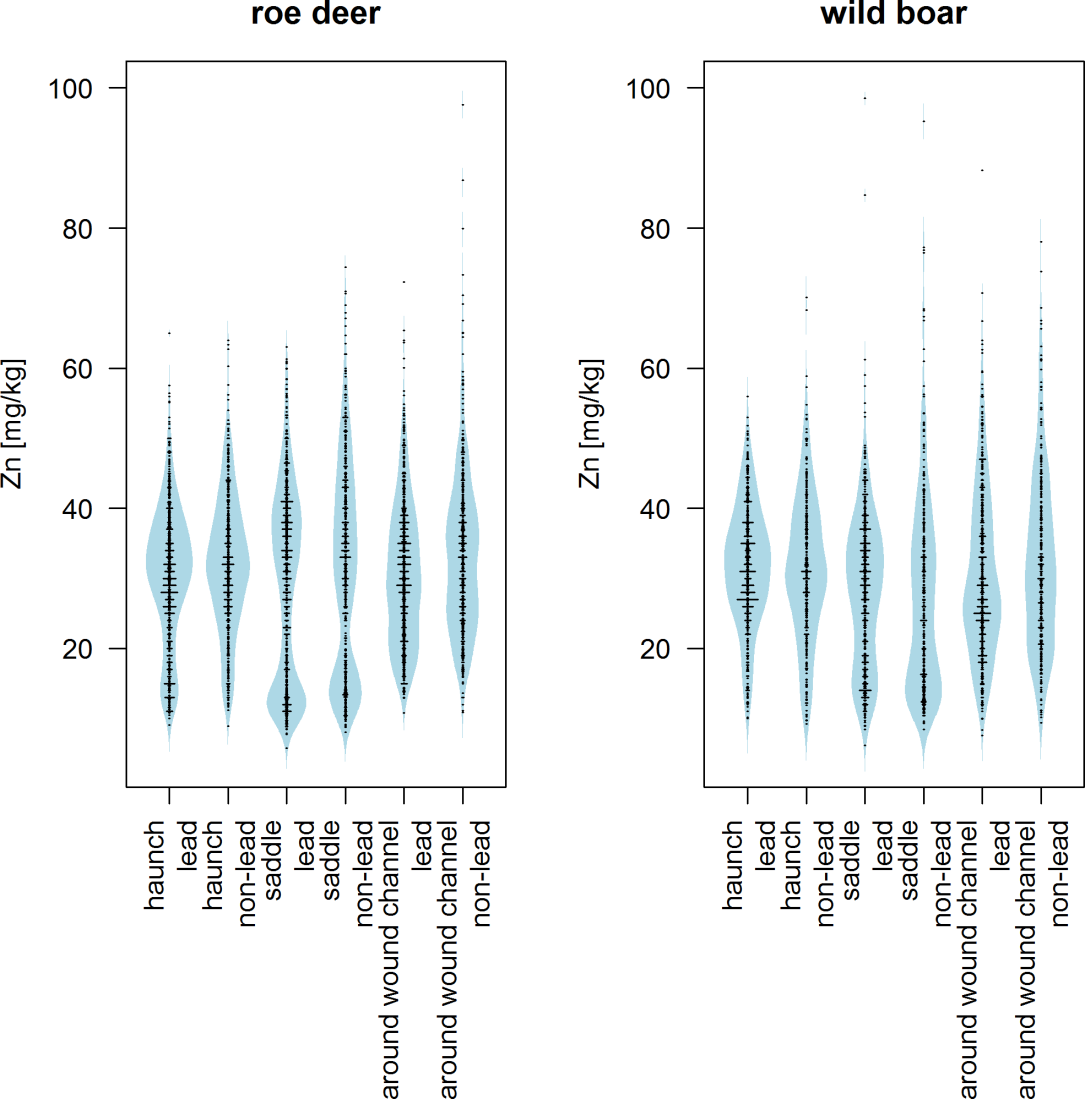
Content in different edible parts of roe deer and wild boar by bullet material (lead, non-lead).

The zinc content in the samples of roe deer as well as in those of wild boar varied considerably, but extreme values were only sporadically found (Fig 2 and S2 Fig).

The zinc content in roe deer samples from the area close to the wound channel was significantly higher when using non-lead ammunition compared to lead ammunition (Mann-Whitney U test, p<0.0001). In addition, the zinc content in samples from the saddle was significantly higher when using non-lead ammunition (Mann-Whitney U test, p = 0.006), but the median values were only slightly different. This difference can be seen by looking at the 95th percentile (Table 3), as well as overall distribution (Fig 2). Regardless of the type of ammunition, the roe deer samples from the area close to the wound channel were not significantly different from those from the haunch or saddle (Friedman test; non-lead: p = 0.281, lead: p = 0.149, respectively).

**Table 3:** Zinc content in hunted roe deer, wild boar and red deer (mg/kg).

Sample	Bullet	N	Mean^a^	Median	95th^b^	Maximum	P
Roe deer, haunch	Lead	745	30.574	31.660	44.640	65.000	0.089
	Non-lead	509	31.946	32.000	48.000	64.000	
Roe deer, saddle	Lead	745	28.842	31.324	50.000	63.000	0.006
	Non-lead	509	31.348	31.770	55.800	131.584	
Roe deer, around wound channel	Lead	745	30.532	29.719	48.000	72.296	<0.0001
	Non-lead	509	33.649	32.870	53.624	138.000	
Wild boar, haunch	Lead	514	31.700	32.029	45.700	56.000	0.397
	Non-lead	340	31.358	31.000	49.407	70.073	
Wild boar, saddle	Lead	514	28.266	29.000	45.000	98.521	0.049
	Non-lead	340	27.646	25.975	52.168	95.202	
Wild boar, around wound channel	Lead	514	30.406	28.410	52.000	88.232	0.027
	Non-lead	340	32.360	30.919	55.955	78.036	
Red deer, haunch	Lead	64	33.965	35.216	43.225	52.642	0.302
	Non-lead	26	35.850	36.373	52.410	57.510	
Red deer, saddle	Lead	64	35.371	37.486	53.010	58.990	0.689
	Non-lead	26	35.134	31.569	63.580	74.640	
Red deer, around wound channel	Lead	64	32.992	31.450	48.030	70.457	0.715
	Non-lead	26	34.110	32.575	48.417	67.933	

^a^ Arithmetical mean.

^b^ 95^th^ percentile.

In 171 roe deer samples, the zinc content was above 50 mg/kg (101 of these samples were shot using non-lead ammunition). Of these 171 roe deer samples, 129 samples were bone hits (non-lead: 79 samples, lead: 50 samples).

Samples of wild boar also had significantly higher zinc content in the area close to the wound channel when using non-lead ammunition (Mann-Whitney U test: p = 0.027).

The zinc content of samples from the saddle of wild boar was significantly higher when using lead ammunition as compared to non-lead ammunition (Mann-Whitney U test, p = 0.049). When comparing the subsamples from wild boar shot with non-lead ammunition, the zinc content of samples from the area around the wound channel were significantly higher than those of the samples from the saddle (Wilcoxon signed-rank test: p<0.0001). The zinc content of samples from the area close to the wound channel were also higher than those of the haunch, but they did not differ significantly (Wilcoxon signed-rank test: p = 0.591). When lead ammunition was used, the zinc content in samples from the area close to the wound channel was lower than the zinc content of samples from the haunch (Wilcoxon signed-rank test: p<0.0001). The zinc content of samples from the area close to the wound channel and from the saddle were not significantly different (Wilcoxon signed-rank test: p = 0.048).

The zinc content of 111 wild boar samples was above 50 mg/kg (of these 63 came from non-lead shot animals). Furthermore, 78 of these samples were from animals killed by bone hits (non-lead: 48, lead: 30).

Just as for the copper content, the zinc content of red deer samples showed no significant differences between non-lead and lead ammunition.

When comparing samples of roe deer and wild boar, a significant difference in their zinc content can only be seen for samples of the saddle when using non-lead ammunition. The zinc content of samples from the saddle of roe deer is significantly higher than those of wild boar (Mann-Whitney-U-test, Table 4).

**Table 4:** Differences in zinc content of different tissues from roe deer and wild boar by bullet material (Mann-Whitney U test).

Sample	Bullet	Species	N	Mean^a^	Median	P
Haunch	Lead	Roe deer	745	30.574	31.660	0.1330
		Wild boar	514	31.700	32.029	
	Non-lead	Roe deer	509	31.946	32.000	0.3360
		Wild boar	340	31.358	31.000	
Saddle	Lead	Roe deer	745	28.842	31.324	0.3040
		Wild boar	514	28.266	29.000	
	Non-lead	Roe deer	509	31.348	31.770	<0.0001
		Wild boar	340	27.646	25.975	
Around wound channel	Lead	Roe deer	745	30.532	29.719	0.3330
		Wild boar	514	30.406	28.410	
	Non-lead	Roe deer	509	33.649	32.870	0.0970
		Wild boar	340	32.360	30.919	

^a^ Arithmetical mean.

## Discussion

One of the aims of the LEMISI project was to determine possible differences in the copper and zinc content in game meat of the examined species when using lead or non-lead ammunition for hunting. Both types of ammunition contain copper and zinc. Whereas non-lead bullets are mainly copper-zinc alloys with partly differing copper content, many lead-based bullets used for hunting are surrounded by a tombac jacket, which has a high copper (>80%) and zinc content. For both metals, variations in amount could be observed for lead and non-lead ammunition.

The maximum residue level (MRL) for copper permitted in food of animal origin from pigs, cattle, sheep, goats, horses, poultry and other farm animals is 5 mg/kg (fresh weight) according to regulation (EC) No 149/2008 and the amending regulation (EC) No 396/2005. This regulation applies to all residues of pesticides, veterinary drugs, or biocides in or on food and feed of plant and animal origin. For wild game meat (i.e. the meat after removal of trimmable fat) the permitted residue level so far has been 0.01 mg/kg, which corresponds with the lower level of detection. This is because since spring 2013 “game meat” has been listed under “other terrestrial animal products”in Annex I to regulation (EC) No 212/2013 and the amending regulation (EC) No 396/2005 and no residue value has been derived based on natural content up to now.

In order to account for the natural background levels of copper in game meat (as a result of environmental uptake mainly through feeding), Germany–in its role as “evaluating member state”—proposed a residue level for copper in game meat of 4 mg/kg [23]. The proposed value is derived from German food monitoring data [24], and incorporates the 95th percentile of the determined copper content. EFSA found that the contribution of the proposed MRL to total consumer exposure to copper was negligible. It amounts up to 0.7% of the Acceptable Daily Intake (ADI) of an adult [23]. This fact recommends the setting of the MRL at 4 mg/kg for copper compounds in wild game in order to cover the natural background level of copper observed in the survey conducted in Germany in 2012. It should be noted that the game meat examined for the monitoring had been shot using lead ammunition.

The maximum residue levels mentioned above can be used as general guidance since the results obtained within the scope of the LEMISI-project show that copper is not evenly distributed in the game meat. The data indicate that an exceedance of the maximum residue levels for copper in game meat cannot be excluded and that the variance of the copper content detected is rather large. As shown in Table 1, the maximum residue level was exceeded, in some cases multiple times, in all examined subsamples (i.e. haunch, saddle, meat close to the wound channel) when using either lead or non-lead ammunition for hunting. One sample of roe deer (saddle, non-lead ammunition) had a copper content of 37.5 mg/kg, and one sample of wild boar (saddle, lead ammunition) had a copper content of 110.0 mg/kg.

The results of the copper content in different meat samples do not present a consistent picture. Regarding the 95th percentile, it can be seen that the copper content is slightly higher in the area close to the wound channel than in the saddle or haunch when using non-lead ammunition for hunting wild boar. On the other hand, the copper content measured in roe deer samples of the area close to the wound channel is lower than in samples of haunch and saddle when using either non-lead or lead-based ammunition. However, some of these contradictory findings for copper and zinc could also be a result of the sample size, which may not have been sufficient for some of the subgroups analysed, even though overall it was quite a considerable sample size.

**Fig 3:**
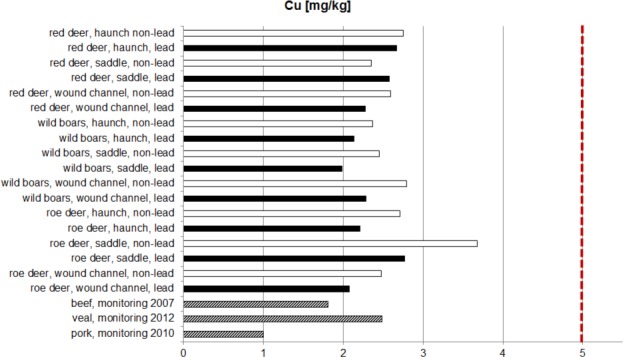
95th percentile copper content of farm animals (German food monitoring program) and game meat (LEMISI) as well as the acceptable maximum residue level of copper in farm animals. Red broken line: 5 mg copper/kg meat.

The median values of the copper content of lead or non-lead shot game meat were relatively close together to a large extent. Lead as well as non-lead bullets result in a comparable entry of copper into the edible parts of the game with only minor differences. Comparing the 95th percentiles of copper content in edible meat of pork, veal and beef with the 95th percentile of the copper content of samples of roe deer, wild boar and red deer, it becomes apparent that pork has the lowest copper content, whereas the percentile values for beef and above all veal are in a range comparable to game meat (Fig 3).

The levels of zinc attributable to the use of lead ammunition are slightly higher in some subsamples as compared to the levels of zinc when shooting with non-lead ammunition. This could be explained by the composition of the bullet material and the bullet construction. A major part of lead ammunition contains varying amounts of zinc in the tombac jacket which surrounds the lead core. Depending on the bullet construction, bullet hit and meat characteristics, varying amounts of zinc are released into the game meat. The median values are only slightly different, even though statistically significant differences have been found for zinc contamination when considering the type of ammunition used (lead or non-lead).

It can be concluded that the content of copper and zinc in game meat in this study are roughly comparable to those found in other studies (Table 5). An analysis of wild boar samples in Austria showed slightly lower ranges in the values (0.86 to 1.48 mg/kg) for the copper content [25]. The zinc content, however, is roughly comparable to this study with a range of values form 24.1 to 60.6 mg/kg. In an analysis of wild boar samples (muscle meat) in western Slovakia, similar values to this study were found with an average copper content of 1.61 mg/kg [26]. In contrast, the zinc content was on average significantly lower (arithmetical average: 13.48 mg/kg). The number of examined samples, however, was markedly lower in both cases.

**Table 5:** European studies on copper and zinc content in game meat (mg/kg wet mass). Table according to Ertl et al. 2016 [27], complemented by additional references.

	Reference	Country	Cu				Zn			
			N	Mean	Median	Max	N	Mean	Median	Max
Roe deer	Dannenberger et al., 2013 [28]	Germany	118	2.8		4.2	118	23.5		39.3
	Falandysz, 1994 (study year 1987) [29]	Poland	145	1.8		8.1	145	30		60
	Falandysz, 1994 (study year 1988) [29]	Poland	84	1.7		6.0	84	36		56
	Hermoso de Mendoza Garcia et al., 2011 [30] **	Spain					75	1.56		8.0
Wild boar	Amici et al., 2012 [31]	Italy	75	12.20	11.80	25.17	57	53.21	53.14	80.10
	Bilandzic et al., 2012 [32]	Croatia	31	3.12	1.68	15.3				
	Dannenberger et al., 2013 [28]	Germany	85	1.7		2.3	85	24.0		31.9
	Falandysz, 1994 (study year 1987) [29]	Poland	149	1.7		5.8	149	32		93
	Falandysz, 1994 (study year 1988) [29]	Poland	118	1.5		5.7	118	37		72
	Gasparik et al., 2012 [26]	Slovakia	120	1.61			120	13.48		
	Roślewska et al., 2016 (males) [33]	Poland	8	6.15		6.8	8	61.28		80.60
	Roślewska et al., 2016 (females) [33]	Poland	8	7.5		9.2	8	68.21		106.1
	Sager, 2005 [25]	Austria	14	1.17	1.19	1.48	14	37.3	34.4	60.6
	Strmisková and Strmiska, 1992 [34]	Slovakia	10	1.3			10	41.0		
Red deer	Falandysz et al., 2005 [35]	Poland	82	3.3		6.4	82	39		64
	Falandysz and Jarzynska, 2011 [36]*	Poland	20	3.63	3.3	7.26	20	49.5	46.2	95.7
	Gasparik et al., 2004 [37]	Slovakia	22	2.49		5.34	22	54.76		109.12
	Lazarus et al., 2008 [38]	Croatia	48	3.48	3.02		48	43.4	43.8	67.4
	Sager, 2005 [25]	Austria	21	1.56	1.62	2.25	21	48.5	53.2	63.8

* Wet mass calculated with 67% water.

** Wet mass calculated with 74% water.

There are further factors which can play a role for the entry of metal into game meat. Among these, there are differences in the physical properties of the ammunition used for hunting due to either the bullet construction or the material composition (alloys), which incidentally may also vary within the classification as non-lead or lead ammunition [39]. However, this could not be analysed in detail in this study due to the limited number of bullet constructions and the corresponding–mainly–low number of samples per bullet type. The muscle meat of hunted species differs too: whereas roe deer exhibits a more tender muscle meat, the muscle meat of wild boar is more solid, resulting in smaller or greater resistance to the bullets [40]. This factor also determines the choice of the bullet construction used for hunting. Fragmenting bullets dispense particles to a greater extent while deforming bullets—which mostly “mushroom”—lead to a few bigger fragments in the surrounding game meat, if at all. Beyond this, the hit of the bullet determines the distribution of the bullet particles in the game meat, e.g. after a bone hit. Furthermore, it is possible that the natural background levels (through absorption from soil, plants, water) could also play a role. In this study, however, the background contamination could not be determined for copper and zinc.

For red deer, no difference was observed in copper and zinc content when using lead or non-lead ammunition. It should be kept in mind though that the sample size was significantly lower than that for the other two species.

The copper and zinc content in game meat is comparable to those regularly detected in the meat of farm animals (pork, beef, sheep) or products made from them (Table 6).

**Table 6:** Copper and zinc content in meat of farm animals (mg/kg).

Sample	N	Mean^a^	Median	90th^b^	95th^b^	Maximum	Reference
**Copper in mg/kg**							
Veal		-	0.76	2.40	3.38	10.10	BVL 2001 [41]
Veal	87	1.57	0.50	-	-	33.00	BVL 2012 [42]
Veal muscles only		1.60				2.40	Souci-Fachmann-Kraut* [43]
Beef		1.07	-	1.67	-	-	BVL 2007 (values from 2002) [24]
Beef		0.83	-	1.60	-	-	BVL 2007 [24]
Beef, muscles only		0.87	-	-	-	1.20	Souci-Fachmann-Kraut* [43]
Pork	80	0.69	0.66	-	-	1.82	BVL 2010 [44]
Pork, leg (hind leg)		3.10	-	-	-		Souci-Fachmann-Kraut* [43]
Lamb/ mutton	116	1.00	0.99	-	-	2.95	BVL 2014 [45]
**Zinc in mg/kg**							
Veal		30.00	-	-	-	-	Souci-Fachmann-Kraut** [43]
Beef		50.10	-	65.20	-	-	BVL 2007 (values from 2002) [24]
Beef		56.90	-	80.60	-	-	BVL 2007 [24]
Beef, muscles only		43.00	-	-	-	49.00	Souci-Fachmann-Kraut [43]
Pork, leg (hind leg)		26.00	-	-	-	-	Souci-Fachmann-Kraut** [43]

- Not available.

* In the original literature given as μg/100g edible percentage.

** In the original literature given as mg/100g edible percentage.

^a^ Arithmetical mean.

^b^ 95^th^ percentile.

Copper compounds play an important role as a feed additive in the fattening of pigs and poultry and are therefore brought into the soil via the application of manure with the result that they enter the food chain. Furthermore, copper compounds are used as fertilizers and pesticides. The exposure of the consumers to copper and zinc is determined by the average consumption habits of the general population.

Considering the exposure with copper and zinc by game meat it has to be included that the consumption rate of the German general population is comparatively low, but nevertheless there are consumers with high consumption rates (“extreme consumers”) and correspondingly higher risk by exposure. For the German population, the mean consumption rate of pork is about 40 kg per year, whereas the average male consumer of game meat in Germany eats two portions of 200 g per year and the average female consumer one portion of 200 g per year. Among the high consumers of game meat are men who eat up to 10 meals and women who eat up to five meals of game meat per year. So-called “extreme consumers”, i.e. hunters and their families, eat up to 90 meals of game meat per year [46]. High male consumers of game meat thus consume almost 18 kg of game meat per year, equalling nearly half the amount of pork meat eaten by the average consumer in Germany.

The mean copper intakes of adults and children in EU countries are below the upper intake level (UL) ranging from 1 mg copper per day for 1–3 year olds up to 5 mg per day for adults with the exception of expectant and nursing mothers [7]. The consumption of game meat contributes to the copper intake. If the mean or median values of the copper content in the game meat are considered, then the intake of copper is between 0.2 and 0.5 mg for average consumption. A health risk for the consumer due to an average consumption of game meat with the reported content of copper is therefore unlikely.

The mean zinc intakes of adults and children in EU countries are below the upper intake level (UL). The UL for adults is about 25 mg per day and for children at the age of one to three years 7 mg per day [7]. The consumption of game meat contributes to the zinc intake. If the mean or median values are considered then the intake is between 5.2 and 7.5 mg per day. A health risk for the consumer due to an average consumption of game meat with the reported content of zinc is therefore unlikely.

Since the general population on average eats more meat and/or products of farm animals, the intake of copper through the consumption of these products is much higher than it is through the consumption of hunted game meat–irrespective of whether lead or non-lead ammunition was used for hunting. This only applies, of course, if game meat hygiene measures have been properly applied, i.e. the meat close to the wound channel has been widely cut out and areas with hematomas have also been widely removed.

From the point of view of consumer health protection, a health risk due to the presence of copper and zinc in game meat at typical consumer exposure levels is therefore unlikely due to the comparably low hazard potential of copper and zinc as compared to lead.

## Supporting information

S1 Fig Beanplot of copper content in different edible parts of roe deer and wild boar by bullet material (logarithmic scale). (PDF)

S2 Fig Beanplot of zinc content in different edible parts of roe deer and wild boar by bullet material (logarithmic scale). (PDF)

S1 File Data File. This file (Zip format) contains the data file (both csv and xlsx format) on which analyses were based and a corresponding readme file.
(ZIP)
