# Supplementary figures and images for: Copper and zinc content in wild game shot with lead or non-lead ammunition – implications for consumer health protection
Source: PLoS One. 2017 Sep 21;12(9):e0184946. doi: 10.1371/journal.pone.0184946 (PMC5608235; doi:10.1371/journal.pone.0184946)

## roe deer

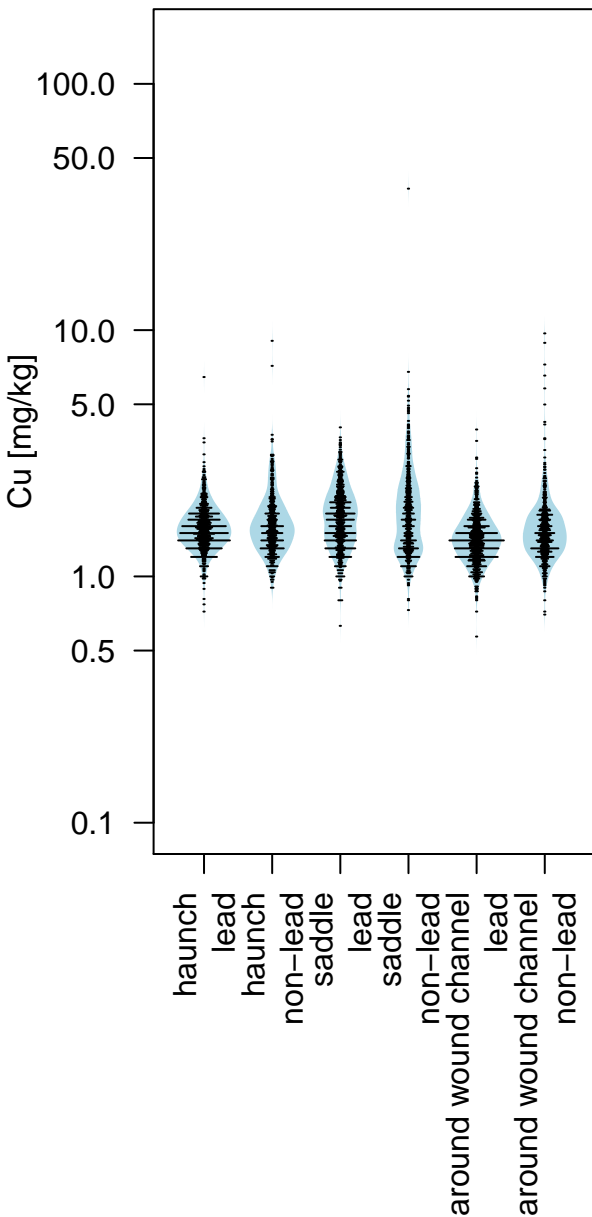

## wild boar

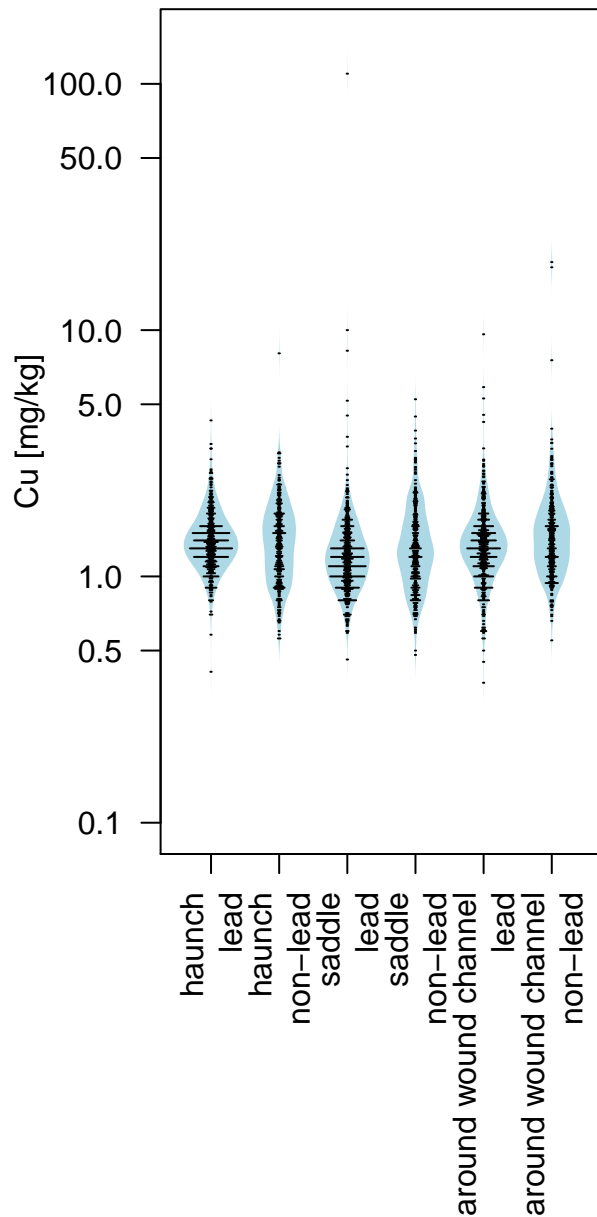

Supplement: S1 Fig — (PDF) [file pone.0184946.s001.pdf]

## roe deer

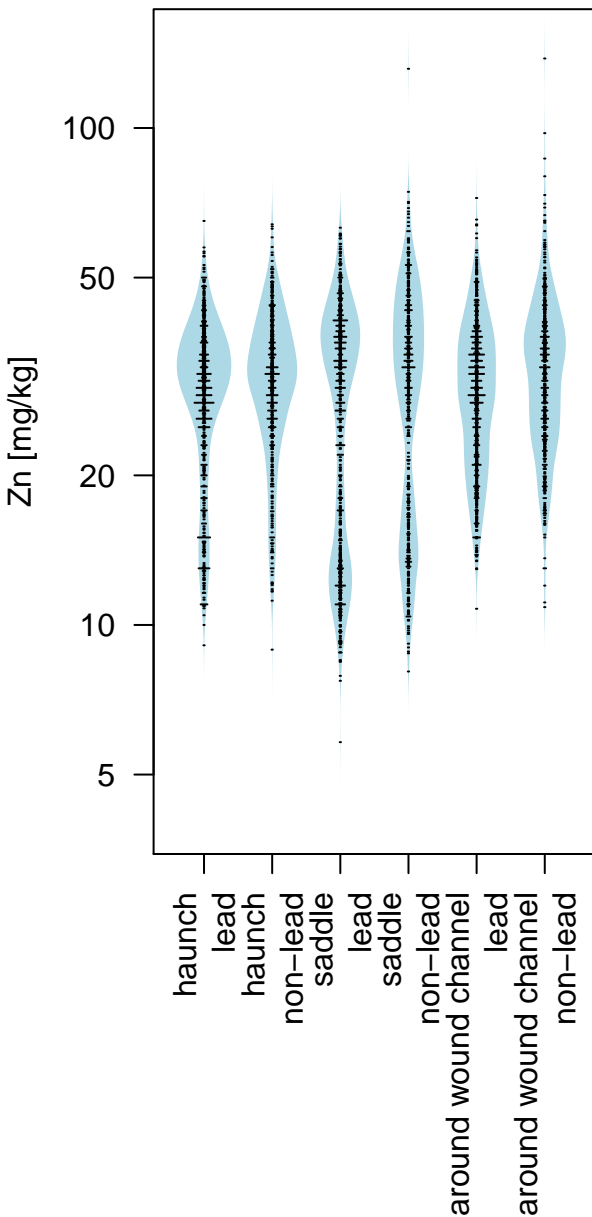

## wild boar

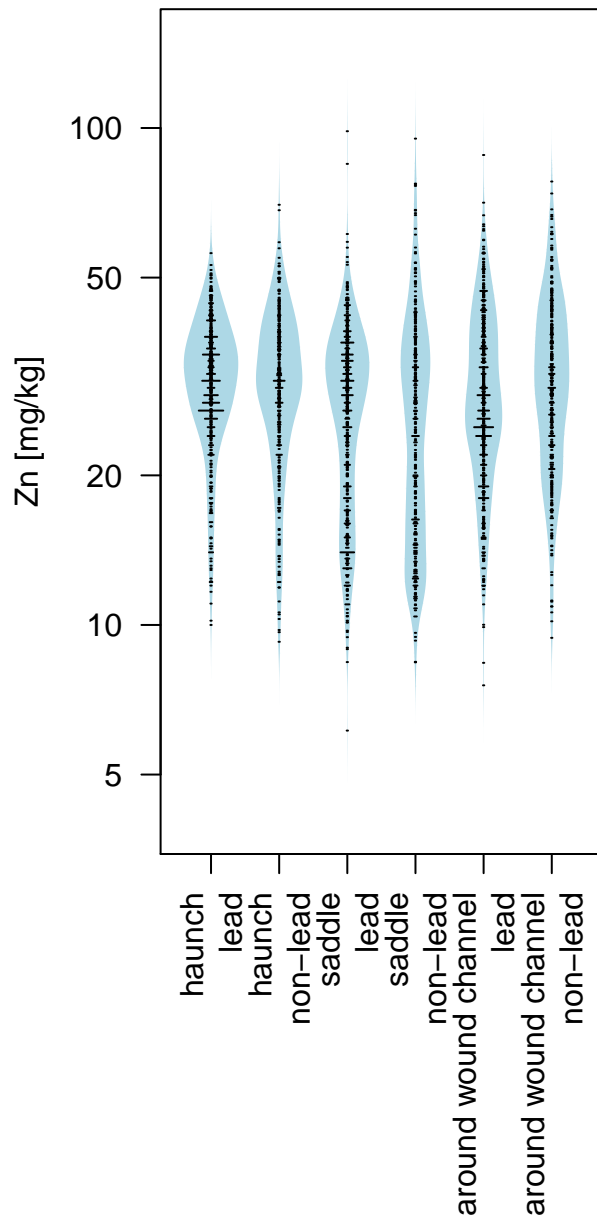

Supplement: S2 Fig — (PDF) [file pone.0184946.s002.pdf]
