# Supplementary material for: Copper and zinc content in wild game shot with lead or non-lead ammunition – implications for consumer health protection
Source: PLoS One. 2017 Sep 21;12(9):e0184946. doi: 10.1371/journal.pone.0184946 (PMC5608235; doi:10.1371/journal.pone.0184946)
Supplement: S1 File — This file (Zip format) contains the data file (both csv and xlsx format) on which analyses were based and a corresponding readme file. (ZIP) [file pone.0184946.s003.zip › Data/Data_description.docx]

Variables in the dataset provided for Copper and zinc content in wild game shot with lead or non-lead ammunition – implications for consumer health protection, by Schlichting D. et al.

| **Variable name** | **Variable label** | **Variable category value & label** |
| --- | --- | --- |
| ID | ID (sample) |  |
| species | Species | 1 roe deer  2 wild boar  3 red deer |
| haunch_cu | Copper content, haunch | *Continuous* |
| haunch_zn | Zinc content, haunch | *Continuous* |
| saddle_cu | Copper content, saddle | *Continuous* |
| saddle_zn | Zinc content, saddle | *Continuous* |
| wound_channel_cu | Copper content, around wound channel | *Continuous* |
| wound_channel_zn | Zinc content, around wound channel | *Continuous* |
| bullet | Bullet material | 1 lead  2 non-lead |
| bone_hit | Bone hit | 1 bone hit  2 other |
